# Evolving insights on how cytosine methylation affects protein–DNA binding

**DOI:** 10.1093/bfgp/elu040

**Published:** 2014-10-14

**Authors:** Ana Carolina Dantas Machado, Tianyin Zhou, Satyanarayan Rao, Pragya Goel, Chaitanya Rastogi, Allan Lazarovici, Harmen J. Bussemaker, Remo Rohs

**Keywords:** epigenetics, DNA methylation, 5-methylcytosine, protein–DNA interactions, DNase I endonuclease, transcription factors

## Abstract

Many anecdotal observations exist of a regulatory effect of DNA methylation on gene expression. However, in general, the underlying mechanisms of this effect are poorly understood. In this review, we summarize what is currently known about how this important, but mysterious, epigenetic mark impacts cellular functions. Cytosine methylation can abrogate or enhance interactions with DNA-binding proteins, or it may have no effect, depending on the context. Despite being only a small chemical change, the addition of a methyl group to cytosine can affect base readout via hydrophobic contacts in the major groove and shape readout via electrostatic contacts in the minor groove. We discuss the recent discovery that CpG methylation increases DNase I cleavage at adjacent positions by an order of magnitude through altering the local 3D DNA shape and the possible implications of this structural insight for understanding the methylation sensitivity of transcription factors (TFs). Additionally, 5-methylcytosines change the stability of nucleosomes and, thus, affect the local chromatin structure and access of TFs to genomic DNA. Given these complexities, it seems unlikely that the influence of DNA methylation on protein–DNA binding can be captured in a small set of general rules. Hence, data-driven approaches may be essential to gain a better understanding of these mechanisms.

## WIDESPREAD ROLE OF DNA METHYLATION

A key epigenetic mechanism in mammals, DNA methylation plays a central role in development [1], specifically in X-chromosome inactivation [2], genomic imprinting [3] and genome reprogramming [4] during early embryogenesis and gametogenesis. DNA methylation suppresses the expression of repetitive sequences [5] and has been implicated in neural development, neurogenesis, synaptic plasticity, learning and memory, brain function, aging and the immune response [6–8]. Chemically, DNA methylation amounts to the covalent addition of a methyl group at the fifth carbon position of cytosine, forming 5-methylcytosine (5mC). As the methyl group is positioned in the major groove, DNA methylation does not interfere with Watson–Crick base pairing [9]. Another chemical modification of DNA is cytosine hydroxymethylation (5hmC) [10, 11], which recent studies suggest may be a novel epigenetic mark that is relevant to development and disease [12–14]. Despite this relationship, the present review focuses on the 5mC modification.

DNA methylation is mediated by a family of well-studied enzymes, DNA methyltransferases (DNMTs), which catalyze the addition of a methyl group to cytosine [15, 16]. In mammalian adult somatic cells, cytosine methylation typically occurs in a CpG dinucleotide context. However, non-CpG methylation is prevalent in embryonic stem cells [17] and has been implicated in neural development [18, 19]. In plants such as *Arabidopsis thaliana*, cytosine can be methylated at CpG, CpHpG and CpHpH sites, where ‘H’ represents any nucleotide other than guanine [20, 21]. Also, in plants, an alternative RNA-directed cytosine methylation pathway exists [22, 23]. Although 5mC has not been detected in yeast or *Caenorhabditis elegans*, and it occurs at a negligible level in *Drosophila melanogaster* [24], some other fungi such as *Neurospora crassa* have a well-characterized methylation system [25].

Considerable attention has recently been given to the functional role of DNA methylation in epigenetic inheritance. Several studies have investigated how DNA methylation patterns can be influenced by developmental and environmental factors, such as parental nutritional exposure [26–29]. Moreover, DNA methylation seems to have an effect on subsequent generations [30, 31].

## DNA METHYLATION AND DISEASE

Normal cell behavior depends on a precise balance between the various nuclear factors and enzymes involved in DNA methylation. Deregulation of this epigenetic mark often affects posttranslational histone modifications and is a contributing factor in different cancers. Aberrant chromatin structure is a common feature in cancer, and numerous comprehensive reviews have linked aberrant methylation to tumorigenesis [32–35]. Two types of DNA methylation changes are observed in cancer: hypomethylation, which is often linked to chromosomal instability and loss of imprinting [36], and hypermethylation, which can lead to transcriptional silencing [37]. A recent study provided evidence for an increased incidence of spontaneous cancers in mice caused by transcriptional suppression through promoter hypermethylation [38]. Studies related to DNA methylation and cancer have broadly focused on the methionine cycle in cancer cells [35], regions with differential DNA methylation patterns in cancer [39], tumor heterogeneity arising from methylation variability across different tumor types [32] and roles of microRNAs [40] and retrotransposons [41] in establishing aberrant methylation patterns.

DNA methylation plays a role in diseases and disorders other than cancer. Several neurodevelopmental or neurodegenerative disorders (e.g. Rett [42], Rubinstein-Taybi [43] and Fragile X syndromes [44], Alzheimer's [45] and Huntington's diseases [46]) and psychiatric disorders (e.g. depression, anxiety, addiction and schizophrenia) have a DNA methylation component [47, 48]. Atherosclerosis has been attributed to DNA hypomethylation [49], and studies have implicated DNA methylation in obesity [50, 51] and cholesterol biosynthesis [52]. Evidence exists for a correlation between abnormal methylation and pathogenesis of the immune system [53]. In particular, hypomethylation of select DNA promoters in T-cells leads to aberrant development, resulting in autoimmune diseases such as lupus [54]. Finally, a role for increased methylation levels in aging and the estimation of age using DNA methylation levels has been suggested [55]. Taken together, these observations leave little doubt about the functional importance of DNA methylation (Table 1).

**Table 1:** Widespread role of DNA methylation in regulation and disease

Biological processes that involve DNA methylation	Examples
Transcription factor binding and nucleosome positioning	[37, 38, 56–60]
Development, differentiation, genomic imprinting and X-inactivation	[1–4]
Cancer	[32, 33, 35–41, 61, 62]
Neurodevelopmental diseases: Rett, Rubinstein-Taybi and Fragile X syndromes	[42–44]
Neurodegenerative diseases: Alzheimer’s and Huntington’s diseases	[45, 46]
Psychiatric diseases: depression, anxiety, addiction and schizophrenia	[47, 48]
Atherosclerosis	[49]
Obesity and cholesterol biosynthesis	[50, 51]
Aging	[14, 52, 55]
Immunity	[8, 54]

Nevertheless, the molecular mechanisms that explain the function of DNA methylation are poorly understood. Therefore, in this review, we analyze the biophysical mechanisms associated with cytosine methylation and how these mechanisms can potentially explain the functional impact of cytosine methylation. In particular, we discuss the recent discovery that CpG methylation increases DNase I cleavage at adjacent nucleotides by an order of magnitude, through narrowing of the DNA minor groove and increasing the Roll angle in CpG dinucleotides [63]. We examine the possible implications of these structural insights for understanding the methylation sensitivity of transcription factors (TFs). More generally, we discuss open questions in this complex, relatively uncharted and evolving field of research.

## DNase I CLEAVAGE IS DEPENDENT ON THE DNA METHYLATION STATE

The classic footprinting endonuclease DNase I has been exploited to probe chromatin structure on a genome-wide scale across many cell types [64]. With the advent of massively parallel sequencing, it has become possible to achieve single-nucleotide resolution. Several of the authors of this review recently characterized the intrinsic sequence biases of DNase I when cleaving naked DNA [63]. When analyzing deeply sequenced DNase I digests of purified genomic DNA, the authors discovered that the rate at which individual phosphodiester bonds were nicked varied dramatically with local sequence context. Specifically, the rates at the most and least cleavable hexamers differed by no less than a factor of a thousand. A later study confirmed this observation and emphasized the importance of considering these strong biases when analyzing *in vivo* DNase I footprinting data at single-nucleotide resolution [65].

**Figure 1:**
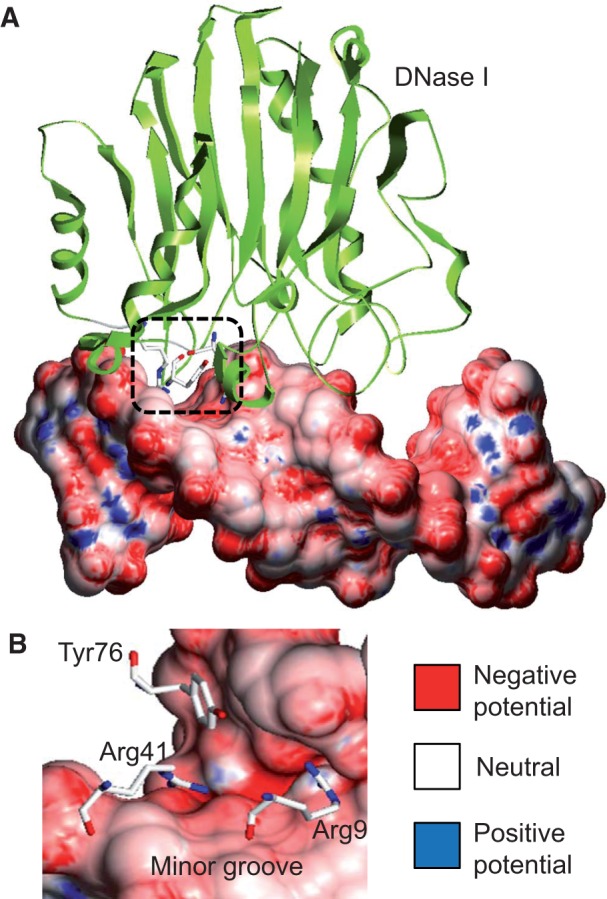
Complex of DNase I endonuclease bound to DNA. (**A**) Cocrystal structure of the complex (PDB ID 2DNJ) illustrates that DNase I binds DNA through contacts to the DNA minor groove. (**B**) A rotated view of the region contacted by DNase I shows that positively charged Arg9 and Arg41 residues recognize the negative electrostatic potential in the DNA minor groove, while Tyr76 stacks with a hydrophobic sugar moiety. Electrostatic potential was calculated for only the DNA molecule taken from the complex at physiologic ionic strength (0.145 M, based on a previously described protocol [66, 67]). Electrostatic potential is shown at the molecular surface of the DNA, using blue for +10 kT/e, red for −10 kT/e and white for neutral potentials.

In addition to the dramatic dependence of the DNase I cleavage rate on the local primary sequence context, Lazarovici *et al.* discovered that direct 5′ cleavage of CpG base pair steps was enhanced by an order of magnitude when the two cytosine bases were methylated [63]. Moreover, they were able to provide a unified quantitative structural explanation for the sequence and the methylation dependence of DNA nicking by DNase I. Briefly, variations in sequence lead to variations in the detailed 3D geometry of the DNA molecule. For each primary sequence, the corresponding DNA duplex assumes a B-form configuration. However, local geometric parameters, such as Roll and minor groove width (MGW), vary over a sufficiently large range to influence the interaction with DNase I. The effect of these DNA shape features on DNase I cleavage is due to the recognition of electrostatic potential in the DNA minor groove through arginine residues (Figure 1) [68].

**Figure 2:**
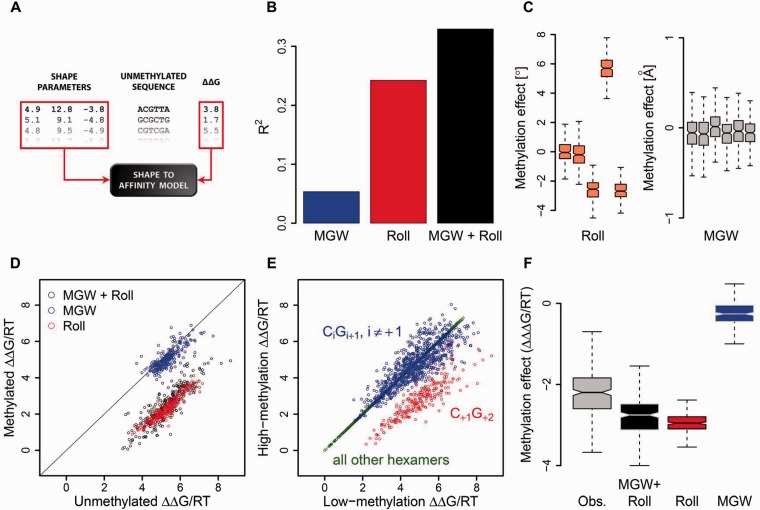
Intrinsic DNA methylation sensitivity of DNase I. This figure illustrates the original analysis performed by Lazarovici *et al.* [63]. (**A**) Schematic diagram illustrating construction of the ‘shape-to-affinity model’ for predicting the binding free energy ΔΔG (logarithm of the relative cleavage rate) from DNA structural features, such as Roll and MGW. (**B**) Fraction of the variance in binding free energy explained by the different variants of the shape-based model when fit to all 256 unmethylated DNA sequences containing a CpG dinucleotide. (**C**) Effect of cytosine methylation on each shape parameter (distribution of difference for Roll and MGW values, along the leading strand of the hexamer), derived from all-atom Monte Carlo simulations [69, 70] of methylated DNA fragments. Five Roll values describe the base pair steps in a hexamer, whereas each of the six base pairs can be assigned a MGW value, as previously described [71]. (**D**) Change in binding free energy for each sequence, predicted from changes in shape features in (C), using the model constructed in (A). (**E**) Empirically observed change in binding free energy obtained from parallel methylation and DNase I profiling on genomic DNA from the same cell line. (**F**) Effect of methylation across all 256 nucleotide sequences, quantified as the change in ΔΔG, as empirically observed and as predicted using Roll, MGW or both.

Figure 2A illustrates the construction of the ‘shape-to-affinity model’ used by Lazarovici *et al.* [63]. Each hexamer sequence is converted into a set of position-specific Roll and MGW values, which serve as the independent (‘predictor’) variables in a multiple linear regression. The negative logarithm of the relative cleavage rate serves as the dependent (‘response’) variable, which can be interpreted as the binding free energy ΔΔG relative to that for the most cleavable sequence (for which, by definition, ΔΔG = 0). Thus, a value of ΔΔG/RT = 1 corresponds to a relative cleavage rate of 37% and ΔΔG/RT = 2 corresponds to a relative rate of 14%, etc. Analyzing the fraction of the total variance of ΔΔG among all unmethylated sequences of type NNN|CGN (where ‘|’ indicates the cleavage site) that can be explained by different variants of the shape-based model (Figure 2B) shows that although Roll is more predictive than MGW, the two variables complement each other.

Lazarovici *et al.* [63] applied computationally intensive Monte Carlo simulations of free DNA to obtain the Roll and MGW values across the DNA molecule [69, 70], which were used as predictors in the shape-to-affinity model. Simulations were performed with or without a methyl group added to each cytosine base of CpG steps in the DNA molecule. This approach enabled the authors to build a shape-to-affinity model from the shape parameters derived from unmethylated sequences of the form NNN|CGN, as well as to predict, for each DNA sequence, the change in binding free energy associated with methylation of the CpG dinucleotide downstream of the cleavage site. Strikingly, the simulations revealed that a change in Roll owing to the substitution of C by 5mC was largely independent of the identity of the other four bases within the NNN|CGN hexamer (Figure 2C). Moreover, a linear model was trained on shape parameters derived from unmethylated sequences. When only the shape changes in the sequences were used to transfer the model to methylated DNA, the model predicted an increased binding free energy that was independent of the sequence context and indicated increased binding in the presence of 5mC (Figure 2D).

The qualitative observation that methylation increases the DNase I cleavage rate by a fixed multiplicative factor (corresponding to the shift in binding free energy) and the quantitative prediction of the magnitude of this effect agreed well with direct empirical observations made by using parallel DNase I footprinting and methylome data for genomic DNA from the same cell line (Figure 2E). Interestingly, Roll seemed to be more useful than MGW for predicting the effect of CpG methylation on binding free energy (Figure 2F). Analysis of the empirical data revealed an ∼9-fold change in cleavage rate (and, presumably, in protein–DNA binding affinity) associated with CpG methylation (median ΔΔΔG/RT = −2.2). However, this value should be interpreted as a lower bound, because it was obtained by constructing hexamer cleavage tables from the top and bottom 20% of genomic locations in terms of observed cytosine methylation levels. Consistent with this finding, comparisons of simulations of strictly unmethylated and fully methylated DNA molecules with a model driven by the empirical cleavage rate for unmethylated sequences [63] predicted a change in the cleavage rate of up to 16-fold on methylation (median ΔΔΔG/RT = −2.8).

## IMPLICATIONS FOR DNA BINDING OF TFS

Mechanisms explaining the change in the DNase I cleavage rate on CpG methylation [63] suggest that 5mC could have an impact on protein–DNA interactions in general, and on the binding specificity of TFs in particular. In the case of DNase I, it is important that two arginine side chains be present in the minor groove (Figure 1). Thus, the magnitude of the enhancement of an interaction based on a similar mechanism is expected to depend strongly on the DNA recognition mechanism used by the specific protein [72–74]. More generally, the hydrophobic methyl group can influence direct contacts in the major groove (base readout), CpG methylation can alter the 3D structure of the DNA binding site (shape readout) and methylated cytosines can modify the nucleosome stability (and, thus, chromatin structure).

Any of these effects can occur in isolation or combination; hence, it is unlikely that the effect of CpG methylation on TF-DNA binding can be summarized in a set of simple rules. Moreover, DNA methylation interacts with other epigenetic marks in a complex manner to affect transcription and regulate gene expression [21, 75]. The exact mechanisms are still not understood, although several have been proposed. TFs might be subjected to a physical barrier created by DNA methylation, which hinders access to TF binding sites (TFBSs). TFBSs that are stably bound by TFs are highly resistant to *de novo* methylation [76]. Mechanical properties of DNA, such as stiffness [77] and strand separation [78], change upon cytosine methylation, with possible effects on TF binding.

## EFFECTS OF DNA METHYLATION ON DNA BASE AND SHAPE READOUT

**Figure 3:**
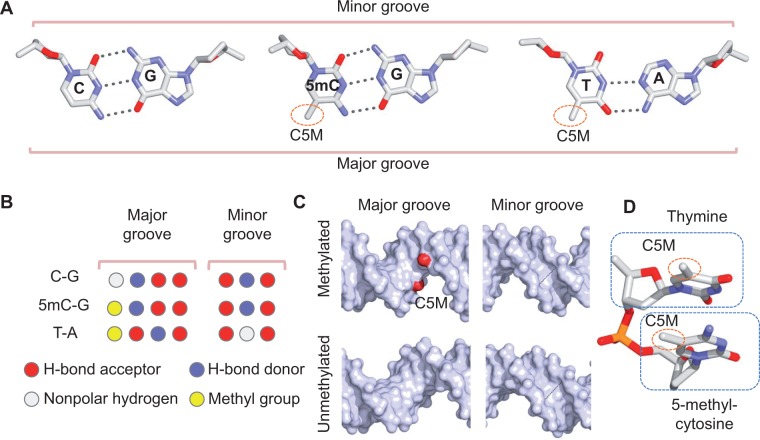
Base and shape readout of methylated DNA. (**A**) Chemical configurations of C-G (left), 5mC-G (center) and T-A (right) base pairs. The methyl group (indicated by the C5M carbon atom) is present at the major groove edge of the 5mC-G and T-A base pairs. (**B**) Signatures of functional groups at the major groove (left) and minor groove (right) edges of C-G (top), 5mC-G (center) and T-A (bottom) base pairs. The methyl group (yellow) changes the signature of functional groups at the major groove edge of the C-G base pair, but base readout at the minor groove edge is not affected. (**C**) Presence of a 5mCpG dinucleotide (C5M carbon atom of the methyl groups shown in red) in methylated DNA (top) can affect the widths of the major (left) and minor grooves (right) compared with unmethylated DNA (bottom) as a function of its sequence context. (**D**) The methyl group of the 5mC nucleotide is in close proximity to the sugar moiety and phosphate group of the adjacent nucleotide in 5′ direction (here, thymine). Structures in this figure are derived from all-atom Monte Carlo simulations of naked DNA, using a previously described protocol [69, 70].

Three major families of methyl-CpG–binding proteins can be distinguished on the basis of the domain structure that they use to interact with DNA: methyl-CpG–binding domain (MBD) proteins, SET and RING-finger associated domain proteins, and Kaiso-like C_2_H_2_ zinc-finger proteins [79–81]. The most intuitive effect of CpG methylation on TF–DNA binding is the addition of a methyl group at the major groove edge of the cytosine base (Figure 3A). The 5-methyl group is present in 5mC and thymine. Thus, the 5mC-G base pair can be contacted through hydrophobic contacts, similar to how thymine is contacted in unmethylated DNA. Cytosine methylation alters functional group signatures in the major groove, but not in the minor groove (Figure 3B). Addition of a methyl group at the major groove edge of 5mC can be specifically recognized through hydrophobic base contacts (base readout).

Of the few crystal structures of DNA oligonucleotides with 5mC bases that have been reported, some of these structures suggest that the steric hindrance of the methyl group in the major groove counters DNA bending and twisting [82]. The presence of a bulky methyl group might lead to a subtle widening of the major groove and, consequently, a subtle narrowing of the minor groove (Figure 3C), owing to the close proximity of the methyl group to the phosphodiester backbone, which might lead to steric hindrance (Figure 3D).

DNA-binding proteins recognize the 3D DNA structure (shape readout) [66, 83], which is highly sequence dependent [84–86]. As discussed above, Lazarovici *et al.* [63] showed that the impact of DNA methylation on DNA structure can explain the methylation state-dependent DNase I cleavage rate. Local DNA shape features were powerful predictors of the effect of cytosine methylation on DNA shape [63]. The effect was strongly sequence dependent in that it only occurred for protein–DNA binding events that led to cleavage of the phosphate immediately 5′ of the CpG dinucleotide. However, the favorable (negative) contribution of methylation to the overall protein–DNA binding free energy was largely independent of the base identity at other nucleotide positions near the cleavage site. Whereas the DNase I cleavage rates of unmethylated CpG-containing sequences varied widely, the fold increase in these cleavage rates because of methylation was the same for all sequences.

Using all-atom Monte Carlo simulations [69, 70], we observed that the Roll angle of CpG dinucleotides consistently increased upon cytosine methylation [63]. This effect occurred for both fully and hemi-methylated CpG steps, as only the size of the increase in Roll depended on sequence context. Roll decreased at the two base pair steps surrounding a CpG dinucleotide, to compensate for the increased Roll at the CpG step. This observation was in agreement with all-atom molecular dynamics simulations, in which the increase in Roll at CpG steps was the most pronounced effect of cytosine methylation on DNA structure [77].

## INSIGHTS FROM STRUCTURES OF PROTEIN–DNA COMPLEXES

Few structures containing 5mC bases have been solved by X-ray crystallography or nuclear magnetic resonance spectroscopy. Several structural studies have demonstrated the importance of the 5mC methyl group for contacts with hydrophobic patches on the protein surface of MBDs [87, 88] or with water molecules [89, 90]. Understanding the role of hydrophobic contacts with the 5mC methyl group in the major groove for methylation state-specific binding ideally requires 3D structures of a protein bound to methylated and unmethylated copies of its DNA target. However, this information is only available for a few TFs, including the zinc-finger proteins Kaiso [91] and Kruppel-like factor 4 (Klf4) [92, 93].

Recognition processes for methylated and unmethylated DNA use similar overall geometries. When Kaiso or Klf4 is in contact with methylated DNA [92, 94], arginine and glutamate form hydrophobic contacts with the cytosine methyl groups. For Kaiso, a hydrophobic pocket built of threonine and cysteine was observed to contact another methyl group [91]. Interestingly, the binding affinities of Klf4 to methylated DNA and to its unmethylated target are similar [92]. In the case of the zinc-finger protein Zfp57, structural information is only available for binding to methylated DNA. The cytosine methyl group of the Zfp57 target site engages in hydrophobic contacts with arginine [95]. Taken together, these data support the hypothesis that hydrophobic contacts with methyl groups merely fine-tune the binding specificities of TFs.

If the presence of a methyl group confer binding specificity, then in principle, 5mC should mimic the presence of a thymine. This possibility has been experimentally proven for the binding site of the lac repressor. In this case, replacement of a thymine by cytosine caused loss of activity, which was restored when thymine was replaced by 5mC [96]. Another example is the complex of the P22 c2 repressor and its operator. In this case, four thymine methyl groups form a hydrophobic binding pocket for a specific valine contact [97]. Contacts with the thymine methyl group were described as intermediates between weak hydrogen bonds and strong van der Waals interactions [98].

**Figure 4:**
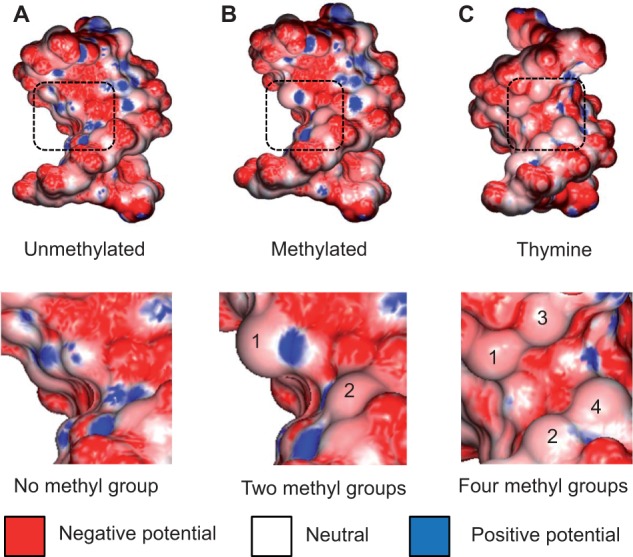
Effect of methyl groups on electrostatic potential of DNA. Hydrophobic methyl groups affect the electrostatic potential in the DNA major groove, as shown for the comparison of the (**A**) unmethylated and (**B**) methylated DNA targets of Klf4 based on cocrystal structures (PDB IDs 2WBU and 4M9E, respectively). A similar effect is observed for a (**C**) DNA target with four thymine methyl groups, which form a binding site for a valine residue of the P22 c2 repressor in the cocrystal structure of the complex (PDB ID 2R1J). The electrostatic potential was calculated at physiologic ionic strength (0.145 M, based on a previously described protocol [66, 67]). The electrostatic potential is shown at the molecular surface of the DNA, using blue for +10 kT/e, red for −10 kT/e, and white for neutral potentials.

A TF or other DNA-binding protein physically interacts via the electrostatic potential at the molecular surface of the DNA [66]. DNA is a polyanion, and its surface is dominated by a negative electrostatic potential. Whereas DNA phosphate groups and bases carry negative charges, the sugar moieties, thymine and 5mC methyl groups of DNA are positively charged [72]. Thus, the electrostatic potential at the molecular surface of these hydrophobic groups is less negative than at the remainder of the DNA surface. We analyzed variations in the electrostatic potential on molecular surfaces of DNA with and without methyl groups using Poisson–Boltzmann calculations at physiologic ionic strength [66]. Comparison of the Klf4 binding sites with and without cytosine methylation [92, 93] clearly showed the impact of the cytosine methyl group on hydrophobic contacts (Figure 4A and B). The same effect was seen for the four thymine-methyl groups, which formed a binding site for a valine of the P22 c2 repressor (Figure 4C) [78].

## LARGE-SCALE BINDING ASSAYS REVEAL METHYLATION-DEPENDENT BINDING

The hypothesis that DNA methylation leads to transcriptional inhibition is still being debated. Despite studies on the effects of cytosine methylation on gene expression [99], the underlying mechanisms are still not understood. A recent high-throughput study of TF binding [56], which combined *in vitro* binding assays with *in vivo* validations, offered a rich perspective on this question. This study used protein microarrays to probe the binding of 1321 human TFs and 210 cofactors to 154 TF binding motifs containing at least one CpG dinucleotide. In general, DNA methylation did not inhibit the binding of TFs from different families. The *in vitro* assay indicated interactions of at least one, and a median of eight, TFs with each of the studied CpG-containing motifs. Moreover, 5mC did not inhibit binding *per se*. A subset of 41 TFs and 6 cofactors from different protein families exhibited specific or nonspecific 5mC-dependent binding. Further analysis of methylated consensus TFBSs with known binding motifs showed almost no correlation, suggesting that cytosine methylation created completely different binding sites for some TFs [56].

## EFFECT OF DNA METHYLATION ON CHROMATIN ACCESSIBILITY AND NUCLEOSOME STABILITY

Another set of putative mechanisms for gene inactivation through DNA methylation involves MBD proteins [100, 101], which establish a positive feedback loop between DNA methylation and specific histone modifications [102]. MBD proteins are capable of acting as histone deacetylase (HDAC)-dependent transcriptional repressors [103]. Repression is achieved by association with methylated DNA sequences [104]. The functional consequences of HDAC–DNMT interactions are still not completely understood, although extensive evidence exists that the catalytically active DNMTs interact with HDACs [61]. DNA methylation enables the DNA binding of MBDs [81], which, in turn, recruit HDACs and other chromatin remodeling proteins. Thus, chromatin becomes compacted and inactive, resulting in gene silencing [62]. DNA methylation can also influence chromatin structure and cause gene silencing [105].

Several studies have addressed the interplay of DNA methylation and nucleosome positioning [57, 58], including one study that used nucleosome mapping in *A. thaliana* [58]. In the latter study, DNase I digestion revealed a consistent 10 bp periodicity, with the minor groove at WW (W = A or T) dinucleotides facing the histone core and the major groove at SS (S = C or G) dinucleotides facing the histone core a half-period (5 bp) away. Interestingly, cytosine methylation of different types (i.e. CpG, CpHpG or CpHpH, where H = A, C or T) showed the same 10 bp periodicity in nucleosomal DNA in phase with the WW dinucleotides, which are regions where the major groove is accessible. This study also revealed a higher methylation level in nucleosome-occupied DNA than in its flanking regions, supporting the argument that DNMTs preferentially bind to nucleosomes. The observed periodicity for all types of cytosine methylation suggested that nucleosomes control access to the DNA for DNMTs [58]. These observations support the hypothesis that DNA is first organized in nucleosomes before being methylated.

Conversely, epigenetic marks (e.g. cytosine methylation) can affect nucleosome positioning. Accurate predictions of nucleosome occupancies are required to study complex regulatory mechanisms. However, most available tools for nucleosome occupancy predictions are knowledge based, fail to account for epigenetic marks and perform poorly on DNA sequences with unknown nucleosome occupancy characteristics. A recently developed physics-based method by Minary and Levitt [59] used the all-atom energy of the 3D nucleosome structure to predict the nucleosome occupancy of a given genomic sequence. Advantages of an *ab initio* method are that it does not require any training, and it can make accurate predictions in the presence of epigenetic marks on a DNA. Minary and Levitt found that the sequence-dependent nucleosome formation energy was anticorrelated with *in vitro* nucleosome occupancy. When all of the cytosine bases in a given genomic sequence were methylated, the dependence of the nucleosome formation energy on the sequence was more moderate. Specifically, the formation of weakly or strongly bound nucleosomes was strengthened or weakened, respectively [59].

The capability of cytosine methylation to moderate the sequence dependence of nucleosome occupancy likely promotes the relocation of nucleosomes to hypermethylated sites within promoter regions. It is interesting to imagine how cytosine methylation could affect the positioning of nucleosomes to change on a fine-grained scale, causing subtle shifts that could expose or block TFBSs. The effect of CpG methylation on nucleosome stability was also evaluated by molecular dynamics simulations [60]. Different nucleosome models were designed with single and multiple CpG steps facing the histone core with either the minor or major groove. Cytosine methylation destabilized nucleosome formation, and multiple steps amplified the destabilization. The stability of nucleosomes correlated with the base-stacking interaction, suggesting that CpG methylation reduced the ability of DNA to tolerate deformation when wrapped around the histone core.

## OPEN QUESTIONS

**Figure 5:**
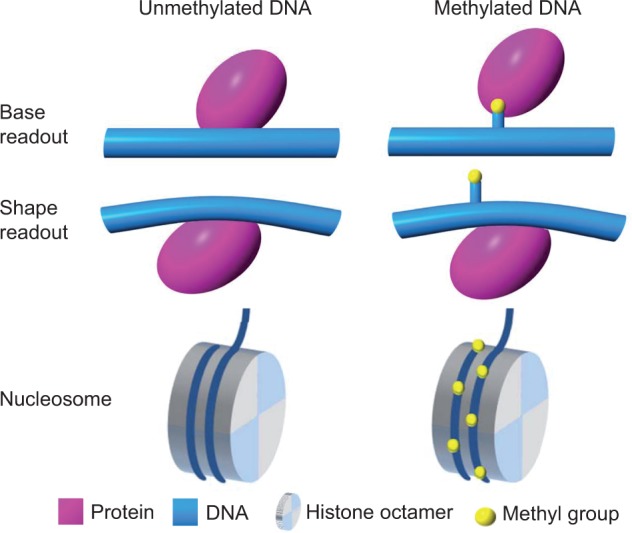
DNA methylation can affect base readout, shape readout and nucleosome stability. Presence of cytosine methyl groups (yellow) can (**A**) affect direct interactions between the protein (purple) and its DNA binding site (blue; base readout), (**B**) cause an indirect effect on DNA structure (blue), which can be recognized by the DNA-binding protein (purple; shape readout) and (**C**) alter nucleosome stability by modifying interactions between the histone core octamer (gray) and the DNA that is wrapped around it (blue).

The biological importance of DNA methylation in development and disease is well established, but a thorough mechanistic understanding of these processes is still lacking. On one hand, the few available experimental structures of TFs bound to methylated versus unmethylated DNA targets suggest that the cytosine methyl group plays only a marginal role in refining hydrophobic contacts, with small effects on binding affinity [91–93]. On the other hand, protein microarray studies suggest that some TFs select novel binding motifs containing methylated CpG dinucleotides [56]. Whereas the exact impact of cytosine methylation on TF binding is an open question, it is known that the DNase I cleavage directly adjacent to CpG dinucleotides strongly depends on the CpG methylation status [63]. Together, these findings suggest that the extent to which TF–DNA binding relies on cytosine methylation likely depends on the readout mode used by the TF under consideration [106]. Base readout can be affected by hydrophobic contacts in the major groove (Figure 5A), and shape readout can vary with the sequence-dependent impact of CpG methylation on DNA structure (Figure 5B).

Another open question regards the effect of DNA methylation on nucleosome positioning and stability (Figure 5C) and, in turn, their impact on TF binding [107]. Some studies have reported generally decreased nucleosome stability on cytosine methylation, which they attributed to the rigidity introduced by methyl groups [77, 60]. Another study suggested that weakly positioned nucleosomes are stabilized, whereas strongly positioned nucleosomes are destabilized [59]. The latter study also proposed a context-dependent effect of cytosine methylation on nucleosome positioning.

Finally, it remains unclear whether the nucleosome core particle provides a scaffold for DNMTs to contact the DNA while bound to the histone octamer [58] or whether the core particle protects DNA from cytosine methylation [108]. Context dependence can probably explain some of these apparently contradictory observations, as suggested by other comprehensive reviews discussing the effect of DNA methylation on nucleosome positioning [109], TF binding [110] and gene regulation [111]. This context may include the DNA sequence environment, cooperativity, chromatin accessibility or other higher-order effects on protein–DNA binding [74, 112]. Understanding how DNA methylation can affect so many biological processes will require further studies and the continued development of new experimental and computational approaches.

**Key points**

- DNA methylation has many known functions, both in normal cell function and disease.
- Mechanisms by which 5mC is recognized by the regulatory machinery of the cell remain largely obscure.
- 5mC readout can occur both directly (by base readout) and indirectly (by shape readout).
- DNA shape changes in response to cytosine methylation could be a general mechanism for modulating protein–DNA interactions.

## FUNDING

This work was supported by the National Institutes of Health (grants R01HG003008 to H.J.B. and R.R., R01GM106056 and U01GM103804 to R.R. and U54CA121852 to H.J.B.) and the Alfred P. Sloan Foundation (to R.R.). The open-access publication charges are defrayed through the National Science Foundation (grant MCB-1413539 to R.R.) and the National Institutes of Health (grant R01HG003008 to H.J.B.).
